# Buddy or burden? Patterns, perceptions, and experiences of pet ownership among older adults in Switzerland

**DOI:** 10.1007/s10433-022-00696-0

**Published:** 2022-04-02

**Authors:** Clément Meier, Jürgen Maurer

**Affiliations:** 1 Faculty of Biology and Medicine (FBM), University of Lausanne, Géopolis, FORS, 1015 Lausanne, Switzerland; 2 Present Address: Faculty of Business and Economics (HEC), University of Lausanne, Internef, 1015 Lausanne, Switzerland

**Keywords:** Perceived benefits, challenges, Human-animal interactions

## Abstract

While interactions with pets may yield significant emotional, social, and physical benefits, taking care of them can also be demanding and experienced as a burden, especially among persons with physical restrictions or economically disadvantaged individuals. This study investigates pet ownership and corresponding perceptions and experiences in a nationally representative sample of adults aged 55 years and older in Switzerland. We use data from a questionnaire on human-animal interactions from 1832 respondents administered during wave 7 (2017) in the Swiss country study of the Survey of Health, Ageing, and Retirement in Europe. Multivariable associations between pet ownership and pet owners’ corresponding perceptions and experiences with respondents’ socio-demographic characteristics were estimated using probit and ordered probit models. Slightly more than one-third of adults aged 55 years and older reported owning a pet. Pet owners reported mostly positive experiences with pet ownership, with women showing higher pet bonding levels than men. Moreover, pet ownership was less common among adults aged 75 and older and individuals living in apartments. At the same time, older pet owners aged 75 and above, pet owners living in apartments, and pet owners without a partner reported more positive perceptions and experiences of owning a pet. These findings suggest that promoting pet ownership may help individual well-being and feelings of companionship, especially among women, older adults, and individuals without a partner but also points toward potential selection effects into pet ownership. Financial costs of pet ownership appear to be an important challenge for some older pet owners, notably those with relatively low levels of education and more limited financial resources.

## Introduction

Population aging is a truly global phenomenon, and Switzerland is no exception. The proportion of the world’s population over 60 years of age will nearly double from 12% in 2017 to 22% in 2050, and the share of persons aged 60 and older in Switzerland is projected to increase from 24.1% in 2017 to 35% in 2050 (United Nations et al. 2017). Ensuring high levels of well-being in the growing population group of older adults is, therefore, an important policy priority both globally and in Switzerland, as highlighted, i.a., by the World Health Organization’s (WHO) “Healthy Ageing Framework” (Beard et al. 2016) and the corresponding global and country-specific action plans for the ongoing “Decade of Healthy Ageing 2020–2030” (Gfs bern 2020; Rudnicka et al. 2020). These efforts aim at identifying potential avenues to address key challenges for healthy aging to help maintain high levels of functioning, meaningful relationships, and high levels of subjective well-being among older adults, as well as to reduce common challenges associated with older ages such as loneliness, social isolation or economic struggles with making ends meet. This study thus investigates pet ownership as well as corresponding perceptions and experiences in a nationally representative sample of adults aged 55 years and older in Switzerland to assess the potential role of pets for healthy aging in Switzerland.

Promoting pet ownership is commonly seen as a promising approach to improve individuals’ well-being in later life, as pet ownership has been shown to be associated with increased physical activity (Serpell 1991), better health (Anderson et al. 1992), reduced loneliness (Barker and Wolen 2008), and a greater ability to successfully cope with stressful life events (Siegel 1990). Pet owners often consider their pets as friends, providing support and motivation in their daily lives (Knight and Edwards 2008). Animal companions can be a source of life satisfaction and positive emotions with recreational time spent with pets. Pets may also be instrumental in helping to lift depressive moods and reducing stress (McNicholas et al. 2005; Rieger and Turner 1999; Turner et al. 2003). These findings strongly suggest that pet ownership may be an important source of happiness and emotional wellbeing at older ages.

Since pet owners have to take care of their pets every day, these obligations regarding their companion animal may provide them with a reason to get out of bed and help them generate routines and structure their day. Activities such as grooming or feeding a pet can increase older adults’ physical skills and encourage them to become more active (Serpell 1991). Previous research showed that dog owners were more frequently engaged in moderate or vigorous exercise, spent more time walking, and had a less sedentary lifestyle than non-dog owners (Coleman et al. 2008; Curl et al. 2016; Dall et al. 2017). In addition, Levine et al. (2013) documented lower total cholesterol levels and significantly lower triglyceride levels among pet owners than individuals without pets due to these higher levels of active mobility. Furthermore, medication use for heart diseases or sleeping difficulties and numbers of doctor visits appear to be significantly lower among older pet owners than older adults without a pet (Headey 1999). Interactions with pets may reduce blood pressure as touching pets can have a calming effect (Allen 2002; Friedmann et al. 1980). Compared to non-pet owners, the risk of cardiovascular diseases appears significantly lower among pet owners as they often have more active lifestyles and experience less stress, better social integration, and higher levels of well-being (Chowdhury et al. 2017).

Pets can also help fulfill some basic social needs of their owners, such as emotional closeness, attachment, and social inclusion (Enders-Slegers 2000; Kurdek 2009). Pets are often perceived to listen without judgment and provide unconditional affection, which can help older adults maintain or increase their self-esteem and self-confidence. Pets may provide social support with similar effects as human–human relationships and give a sense of purpose by making older adults feel responsible for their pets and, therefore, useful (Antonacopoulos and Pychyl 2010; Raina et al. 1999). Pet ownership may also affect older adults’ relationships with other humans as pets sometimes act as facilitators of social interactions. Pet owners are significantly more likely to get to know people in their neighborhood than non-pet owners (Wood et al. 2005, 2007, 2015). In many cases, pets can serve as an ice-breaker or a neutral topic to start conversations. Therefore, having a pet reduces the risk of being socially disconnected from others and may improve older adults’ mental health by attenuating feelings of loneliness (Anderson et al. 2008; Stanley et al. 2014). Finally, pets can also help to deal with stressful life events by bringing support and companionship to older adults in the face of adversity (Edney 1995). Overall, existing evidence points toward numerous potential benefits of pet ownership among older adults that could help address some of the most important challenges of old age and, thereby, facilitate healthy aging and help maintain high levels of well-being during later life.

Yet, having to take care of pets may also result in new everyday-life challenges and stresses for older pet owners, such as added time demands to groom and mind the pets (Chur-Hansen et al. 2008), potential stresses of being around very active or demanding pets (Bayliss et al. 2007) or increased financial pressures associated with pet ownership (Chur-Hansen et al. 2008), which may offset some of the aforementioned benefits of pet ownership for older adults. Owning a pet implies many responsibilities, which require energy and skills that may be challenging to some (Chur-Hansen et al. 2008). Pets may also represent a direct health hazard, as pets can potentially transmit diseases or infectious agents, trigger allergic reactions, or display behaviors that may put their owners at risk. Even if the risk of catching an infection from a pet is relatively low and can be reduced by vaccinating the pets and employing proper hygiene practices, pet-related allergies are fairly common and can increase cleaning demands (Bayliss et al. 2007). Also, while relatively rare, incidents involving animals can sometimes lead to injuries in pet owners, such as when pets bite or push owners on the floor (Mallon 1992). Regarding potential mental challenges, losing a pet can be a traumatic experience and lead to anxiety, depression, and loneliness (Smith 2012). Owning a pet can also be expensive, especially when the animal has an accident or is sick, requiring veterinary care (Chur-Hansen et al. 2008). Finally, taking care of pets can be very time demanding. In some circumstances, pet ownership may even lead to foregone healthcare if, say, pet owners refuse potentially needed hospital treatments in order to stay with their pets (Arhant-Sudhir et al. 2011). Hence, despite the widely presumed benefits of having a companion animal, pet ownership may also result in important challenges and stresses that could potentially harm the well-being of older pet owners.

Given the above potential benefits and challenges associated with pet ownership at older ages, assessing pet ownership and the pet owners’ actual perceptions and experiences of pet ownership can help highlight key opportunities and challenges for policies and interventions to promote and facilitate pet ownership among older adults. What is more, exploring the associations of pet ownership and older pet owners’ perceptions and experiences of pet ownership with their socio-demographic characteristics and life circumstances allows us to identify specific groups of individuals that may especially benefit from or be more challenged by different aspects of pet ownership at higher ages. Despite the enormous potential benefits of pet ownership for older adults, there are, to the best of our knowledge, no existing nationally representative studies to date that comprehensively describe the socio-demographic patterns of pet ownership among older adults in Switzerland along with pet owners’ perceptions and experiences including potential challenges of having a companion animal in their household. To address this gap in the literature, we use Swiss data from the 2017 Survey of Health, Ageing and Retirement in Europe (SHARE) to document patterns of pet ownership as well as different perceived benefits and challenges of having a companion among older pet owners.

## Data and methods

### Sample

Our analytical dataset combines questions on pet ownership from a self-administered paper-and-pencil drop-off questionnaire fielded to respondents of wave 7 with socio-demographic information from different waves of the Swiss component study of SHARE obtained from in-person interviews (Börsch-Supan 2022). SHARE is a biennial population-based longitudinal study of Europeans aged 50 years and older that started in 2004 (Börsch-Supan et al. 2013). SHARE collects information on health, socioeconomic status, and social or family networks of targeted respondents and their partners in 27 European countries and Israel. SHARE wave 7 included 2,402 respondents in Switzerland, either as targeted respondents or partners. Among those, 2,282 individuals also completed the drop-off questionnaire for a conditional completion rate of 95%. We restricted our analysis to respondents over the age of 54 because the last sample refreshment for SHARE Switzerland only took place in 2010, such that SHARE wave 7 is not representative of the initial target population of adults aged 50 and older. After deleting all observations with missing information on at least one item used in the analysis, 1,832 individuals were included in our study.

### Measures

**Pet ownership** The drop-off questionnaire assessed pet ownership based on the question “Do you currently have one or more of the following pets in your household?” with permissible non-mutually exclusive answer categories for “dog”, “cat”, “bird”, “fish”, and “others, please specify”. Write-in responses to the “other, please specify” category comprised various small animals such as hamsters, rabbits, guinea pigs, turtles, or snakes, but also farm animals such as chicken, sheep, goats, horses, or cows. Given our interest in pets rather than animal ownership more broadly, we did not classify four individuals who reported to own only farm animals as pet owners.

**Perceptions and experiences of pet ownership** Pet owners were asked to evaluate their perceptions and experiences of pet ownership based on a list of 15 statements (I love having my pet(s) around; I love my pet(s), My pet(s) give me companionship; It is very expensive to take care of my pet(s); I love to take care of my pet(s); My pet(s) is/are my friend(s); I talk to my pet; My pet(s) add to my happiness; I often play with my pet(s); I talk to others about my pet(s); My pet(s) makes me go outside more frequently; My pet(s) help me to engage with other people; My pet knows how I feel about things; My pet(s) go(es) on my nerves; It is very hard work to take care of my pet(s)). Respondents evaluated their level of agreement on a five-point Likert scale (1 = strongly disagree, 2 = disagree, 3 = neither agree nor disagree, 4 = agree, 5 = strongly agree). Some of these statements came from the Health and Retirement Study’s (HRS) 2012 human-animal-interaction module while others were created by the Swiss SHARE country team based on the literature. The list includes statements on positive attitudes and behavior toward pets as well as negative items concerning the cost and work necessary to take care of a pet. We created a pet bonding score constructed by combining all the items in the analysis. The score adds each answer from the five-point Likert scale for the positive items and subtracts the response from the negative answer. We then normalized the score, the maximum possible value of the score is 60, and the minimum is 0.

**Socio-demographic covariates** To assess socio-demographic differences in pet ownership and corresponding perceptions and experiences, our statistical models include information on gender (0 = male, 1 = female), age group (55–64 years, 65–74 years, 75 + years), and education level, which was grouped into three categories based on the International Standard Classification of Education (ISCED) of 2017 (low = ISCED levels 0–1–2, secondary = ISCED levels 3–4, tertiary = ISCED levels 5–6). We also used information on language regions based on the language of the questionnaire (German, French or Italian). Our measure of partnership status considered all types of partnership rather than just focusing on formal marriage (0 = has a partner, 1 = has no partner). Respondents’ work status was also assessed (0 = not working, 1 = working). Respondents’ perceived financial situation was measured based on the question: “Is your household able to make ends meet?” with permissible answers being recoded into three groups (1 = easily, 2 = fairly easily, 3 = with difficulty). We merged the two highest categories “with some difficulty” and “with great difficulty” of the original question format into one new category “with difficulty” due to the relatively low proportion of respondents answering “with great difficulty” (1,85%). We also included information on individuals living environment into our models, namely whether they lived in an urban or rural area (0 = urban, 1 = rural) and their type of housing (0 = apartment, 1 = house).

**Health covariates** We used two variables to assess respondents’ health status: self-rated health and an indicator for depression symptoms. For parsimony, the original five-point Likert scale to measure self-rated health (5 = excellent, 4 = very good, 3 = good, 2 = fair, 1 = poor) was re-coded by combining the two outer categories to obtain a three-point scale (1 = poor/fair health, 2 = good health, 3 = very good/excellent health). An indicator for the presence of depressive symptoms (0 = no depression, 1 = depression) was created using depression, pessimism, suicidality, guilt, sleep, interest, irritability, appetite, fatigue, concentration, enjoyment, tearfulness items forming the EURO-D scale with a cutoff of 3.

### Statistical analysis

We used weighted proportion estimation to assess the relative frequencies of all variables used in our analysis. Specifically, to obtain descriptive statistics that are representative of the population of interest, we used the cross-sectional weights provided in the SHARE data to calibrate the sample. Associations of overall pet ownership and ownership of specific types of pets with respondents’ socio-demographic characteristics were based on weighted multivariable probit models with corresponding results being reported in terms of average marginal effects (AME). Perceptions and experiences of pet ownership among respondents who reported owning a pet were assessed using weighted proportion estimation. In contrast, their corresponding partial associations with pet owners’ characteristics and life circumstances were based on weighted multivariable ordered probit models, whose results are reported in terms of the estimated coefficients on the underlying latent variables. All estimations were performed using STATA/SE 17.0 software (STATA Corporation, College Station, TX). The standard errors for our model estimates were clustered at the household level, as some respondents are partners from the same household, which may lead to unobserved dependencies across these observations.

## Results

Table 1 presents the key characteristics of our weighted analytical sample. 52% of our sample were women, and the mean age of our respondents was 69.58 years old (SD = 8.81). Most respondents had a secondary education degree (64%). 75% were German speakers, with 23% being French and 2% being Italian speakers. 79% of respondents had a partner, and 40% of respondents were working. Concerning respondents' economic status, the majority of the individuals reported that it was “easy” (57%) or “fairly easy” (30%) for them to make ends meet. 55% of our study respondents lived in houses, and 58% lived in rural areas. Most respondents reported being healthy, with only 19% indicating fair or poor health. Concerning mental health, 83% of the sample did not suffer important depressive symptoms as measured by the EURO-D scale. The weighted proportion of older adults who owned at least one pet was 34%. Concerning the type of pets, 70% owned cats, followed by 26% having dogs, with smaller fractions possessing other kinds of pets such as fish or birds.

**Table 1:** Characteristics of the study population, adults aged 55 + , SHARE Switzerland, 2017

	Characteristics of the sample *n* = 1,832			Characteristics of pet owners *n* = 529		
	Unweighted	Weighted		Unweighted	Weighted	
	Obs	%	CI	Obs	%	CI
Pet ownership						
Non-pet Owner	1,289	66	[62.9–69.6]			
Pet owner	543	34	[30.4–37.1]			
Type of pets						
				Dog owner 139	26	[20.4–32.0]
				Cat owner 360	70	[63.7–75.8]
				Other 30	4	[2.6–6.5]
Pet bonding score						
				Mean: 44,0		Std. dev: 9.5
				Min: 10		Max: 60
Gender						
Male	846	48	[45.9–50.3]	237	50	[45.4–54.0]
Female	986	52	[49.7–54.1]	292	50	[46.0–54.6]
Age groups						
55–64 years	615	49	[45.9–52.8]	241	63	[57.8–68.5]
65–74 years	706	29	[26.2–31.3]	208	26	[21.9–30.6]
75 + years	511	22	[19.8–24.3]	80	11	[8.2–13.8]
Partnership status						
Has a partner	1,395	79	[76.2–80.8]	435	85	[80.7–88.1]
No partner	437	21	[19.2–23.8]	94	15	[11.9–19.3]
Language						
German(ch)	1,357	75	[71.8–77.7]	393	75	[69.8–80.3]
French (ch)	434	23	[20.6–26.3]	116	22	[16.9–27.0]
Italian (ch)	42	2	[1.3–2.7]	20	3	[1.7–5.3]
Education						
Low	335	17	[14.5–18.9]	89	13	[10.2–16.3]
Secondary	1,156	64	[60.7–66.7]	333	65	[59.1–70.4]
tertiary	341	20	[17.1–22.4]	107	22	[17.0–28.2]
Make ends meet						
Easily	1,037	57	[53.2–60.1]	296	55	[48.9–61.8]
Fairly easily	573	30	[27.0–33.3]	157	28	[22.9–34.2]
With difficulty	222	13	[10.9–16.0]	76	16	[12.0–21.9]
Employed						
No	1,330	60	[56.1–62.9]	337	49	[43.0–54.6]
Yes	502	40	[37.1–43.9]	192	51	[45.8–57.0]
Living area						
Urban	779	42	[38.9–45.8]	168	32	[25.6–37.8]
Rural	1,053	58	[54.2–61.1]	361	68	[62.7–74.1]
Type of house						
House	998	55	[51.4–58.4]	367	72	[65.9–77.2]
Apartment	834	45	[41.6–48.6]	162	28	[22.8–34.1]
Self-rated health						
Poor/fair health	360	19	[17.0–22.0]	105	20	[15.8–25.2]
Good health	795	43	[39.5–45.6]	212	40	[33.1–44.5]
Excellent health	677	38	[35.1–41.3]	212	40	[35.3–47.2]
Depression						
No	1,556	83	[80.7–85.6]	444	82	[77.1–86.5]
Yes	276	17	[14.4–19.3]	85	18	[13.5–22.9]

Unweighted and weighted number of observations for the whole sample and the sample of pet owners. Data from SHARE wave 7, release version: 7.1.1

**Fig. 1:**
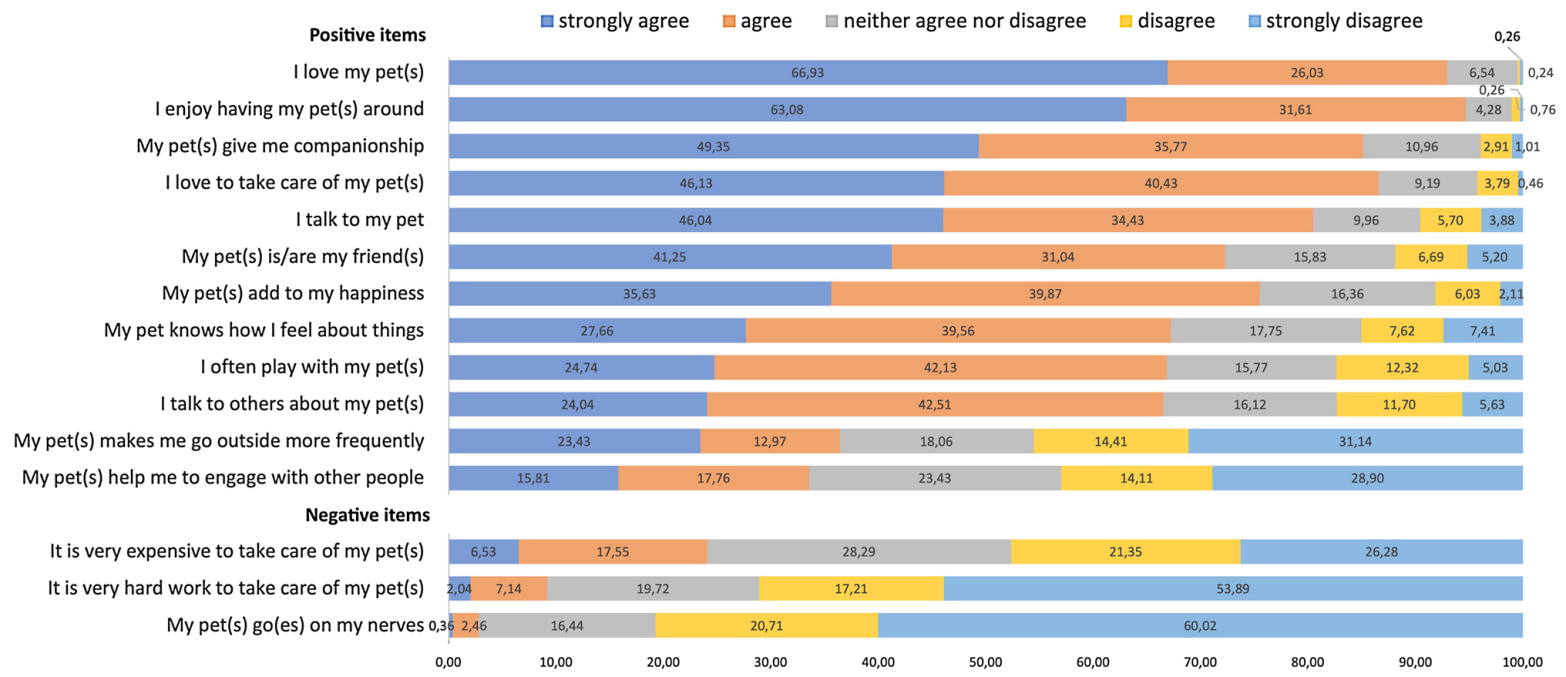
Experiences of pet ownership and companion animal interactions, adults aged 55 + , SHARE Switzerland, 2017, *n* = 529 (*The figure shows the weighted proportions of the perceptions and experiences of respondents on each human-animal interactions item. Data from SHARE wave 7, release version: 7.1.1.)*

The distribution of the 15 statements measuring pet owners' perceptions and experiences of pet ownership presented in Fig. 1 shows that most pet owners agreed or strongly agreed with the statements that they loved their pets (93%) and enjoyed having their pet around (94.7%). Similarly, a large number of respondents indicated that their pets gave them companionship (85.1%) and that they loved to take care of their pets (86.6%). Many pet owners' reported important connections with their pets: 80.5% of pet owners talked to their pets, and 72.3% considered their animal companion a friend. Additionally, 75.5% agreed or strongly agreed that their pets added to their happiness, and 67.2% agreed or strongly agreed that their pet knew how they felt about things. Besides, 66.9% of the respondents stated that they often played with their pets, and 66.5% talked to others about their pets. However, we only found mixed evidence on whether pets made pet owners go outside more frequently or helped them engage with other people. Moreover, pet owners seemed to have mixed opinions on the financial impact of owning a pet: 24% agreed or strongly agreed, 28.3% neither agreed nor disagreed, and finally, 47.6% disagreed or strongly disagreed with the statement that “it is very expensive to take care of my pet(s)”. Only 9.2% agreed with the statement that it was very hard work to take care of their pets, while 71.1% disagreed. Finally, less than 3% of pet owners experienced the feeling of irritability toward their pet(s).

The multivariable probit models from Table 2 present the partial associations of pet ownership with respondents' socio-demographic characteristics both overall and by major pet type. The multivariable results indicate that older adults were less likely to own a pet (AME: − 0.10, *p* < 0.05). Age differences in pet ownership are increasing in age, with respondents older than 75 years being 23 percentage points less likely to own a pet (AME: − 0.23, *p* < 0.001). Compared to the German-speaking region, respondents from the Italian part of Switzerland were more likely to have pets (AME: 0.21, *p* < 0.05). Perhaps somewhat surprisingly, respondents reporting financial difficulties had a larger chance of owning a pet (AME: 0.13, *p* < 0.05). Finally, living in a rural area (AME: 0.08, *p* < 0.05) and a house (AME: 0.20, *p* < 0.001) was associated with a higher likelihood of pet ownership.

**Table 2:** Pet ownership regression, adults aged 55 + , SHARE Switzerland, 2017

	Any pets	Dog	Cat	Other
	AME (SE)	AME (SE)	AME (SE)	AME (SE)
**Gender** (male)				
Female	0.01 (0.02)	− 0.00 (0.02)	0.03 (0.02)	0.00 (0.01)
**Age group** (55–64 years)				
65–74 years	− 0.10* (0.04)	− 0.03 (0.03)	− 0.06 (0.04)	0.00 (0.01)
75 + years	− 0.23*** (0.04)	− 0.09** (0.03)	− 0.15*** (0.04)	− 0.00 (0.01)
**Partnership status** (has a partner)				
no partner	− 0.03 (0.03)	− 0.00 (0.03)	− 0.04 (0.03)	− 0.01 (0.01)
**Language** (German(ch))				
French (ch)	− 0.03 (0.04)	− 0.03 (0.03)	− 0.02 (0.03)	− 0.01 (0.01)
Italian (ch)	0.21* (0.09)	− 0.00 (0.07)	0.09 (0.10)	0.13 (0.09)
**Education** (low)				
Secondary	− 0.04 (0.03)	0.03 (0.02)	0.01 (0.03)	0.02 (0.01)
Tertiary	0.06 (0.05)	0.04 (0.04)	0.03 (0.04)	0.00 (0.01)
**Make ends meet** (easily)				
fairly easily	0.03 (0.03)	0.02 (0.03)	0.01 (0.03)	− 0.01 (0.01)
with difficulty	0.13* (0.05)	0.09* (0.05)	0.15** (0.05)	0.01 (0.02)
**Employed** (no)				
Yes	− 0.00 (0.04)	− 0.03 (0.03)	0.06 (0.04)	− 0.01 (0.01)
**Living area** (urban)				
Rural	0.08* (0.03)	− 0.00 (0.03)	0.12*** (0.03)	− 0.01 (0.01)
**Type of house** (house)				
Apartment	− 0.20*** (0.03)	− 0.09*** (0.03)	− 0.13*** (0.03)	− 0.04*** (0.01)
**Self-rated health** (poor/fair health)				
good health	− 0.05 (0.04)	0.03 (0.03)	− 0.05 (0.04)	0.02* (0.01)
very good/excellent health	− 0.05 (0.04)	0.01 (0.03)	− 0.06 (0.04)	0.01 (0.01)
**Depression** (no)				
Yes	− 0.02 (0.04)	− 0.00 (0.03)	− 0.05 (0.03)	0.00 (0.01)
Observations	1832	1832	1832	1832

Bold values denote statistical significance at * p < 0.05, ** p < 0.01 and *** p < 0.001

This table reports the estimates for the weighted probit models regressing the different types of pet ownership on the covariates. Average marginal effects and standard errors in parentheses have the following significance levels: * *p* < 0.05, ** *p* < 0.01, *** *p* < 0.001. Data from SHARE wave 7, release version: 7.1.1

Table 3 presents the partial associations of the 15 statements measuring the human-animal relationship and the pet bonding score with the individual characteristics and life circumstances of pet owners. Overall, pet owners reported good relationships with their pets; the average pet bonding score was 44 points on a (theoretical) maximum of 60 points. Female pet owners seemed to have had better pet bonding as women were more inclined to agree with the positive items concerning their pet ownership (OLS: 3.79, *p* < 0.001). Pet owners aged 65–74 and 75 + had higher chances of reporting having a good relationship with their pets, with stronger associations for the older age category (OLS: 2.29, *p* < 0.05). Compared to the German-speaking region, pet owners from the French part were less likely to report positive perceptions and experiences with their pets (OLS: − 4.17, *p* < 0.01). The connection between respondents living in an apartment and their pets seemed higher (AME: 0.26, *p* < 0.05). Compared to those with a partner, pet owners without a partner were more likely to report that their pets gave them happiness (AME: 0.47, *p* < 0.01). When considering the financial aspects, older pet owners (AME: 0.33, *p* < 0.05, AME: 0.61, *p* < 0.01), and those with less education (AME: 0.66, *p* < 0.01), more limited financial resources (AME: 0.52, *p* < 0.05), and depressive symptoms (AME: 0.42, *p* < 0.05) were more likely to agree that it is very expensive to take care of their pet(s).

**Table 3:** Human-animal interactions, adults aged 55 + , SHARE Switzerland, 2017

	Pet Bonding	Love	Enjoy	Comp-anion	Care	Talk to	Friend	Happy	Feel	Play	Talk about	Go out	Engage	Expen-sive	Hard- work	Nerves
*Point estimates and standards errors in parentheses*																
**Gender** (male)																
Female	3.79*** (1.02)	0.67*** (0.14)	0.46*** (0.12)	0.44*** (0.13)	0.75*** (0.12)	0.53*** (0.13)	0.27** (0.13)	0.39*** (0.12)	0.35*** (0.13)	0.23 (0.12)	0.39*** (0.13)	0.10 (0.11)	0.14 (0.12)	0.02 (0.12)	− 0.13 (0.13)	0.07 (0.13)
*Age group (55–64 years)*																
65–74 years	2.29* (1,10)	− 0.09 (0.18)	0.11 (0.18)	0.10 (0.15)	0.39* (0.16)	0.32* (0.16)	0.18 (0.14)	0.37** (0.14)	0.19 (0.17)	0.31* (0.14)	0.27 (0.15)	0.32 (0.18)	0.38* (0.15)	0.33* (0.15)	0.08 (0.16)	0.04 (0.17)
75 + years	2.70 (1.45)	− 0.02 (0.23)	0.05 (0.24)	0.36 (0.21)	0.70*** (0.21)	0.55* (0.23)	0.38 (0.20)	0.54** (0.20)	0.36 (0.22)	0.37* (0.19)	0.43* (0.19)	0.19 (0.22)	0.26 (0.22)	0.61** (0.19)	0.32 (0.20)	− 0.17 (0.22)
*Partnership status (has a partner)*																
No partner	2.28 (1.53)	0.30 (0.20)	0.34 (0.20)	0.40 (0.23)	0.28 (0.18)	0.31 (0.22)	0.45 (0.23)	0.47** (0.18)	0.08 (0.18)	0.31 (0.21)	0.17 (0.19)	0.11 (0.17)	0.13 (0.19)	0.17 (0.16)	− 0.13 (0.17)	− 0.18 (0.18)
**Language** (German CH)																
French CH	− 4.17** (1.49)	− 0.29 (0.20)	− 0.23 (0.19)	− 0.13 (0.18)	− 0.25 (0.19)	− 0.49** (0.17)	− 0.17 (0.15)	− 0.16 (0.17)	− 0.32 (0.16)	− 0.29 (0.18)	− 0.33 (0.18)	− 0.27 (0.17)	− 0.28 (0.17)	0.12 (0.15)	0.68*** (0.15)	0.23 (0.16)
Italian CH	0.54 (2.49)	0.19 (0.27)	0.23 (0.31)	0.23 (0.34)	0.47 (0.29)	0.04 (0.35)	0.56* (0.26)	− 0.09 (0.27)	− 0.27 (0.22)	0.06 (0.28)	0.00 (0.27)	− 0.19 (0.24)	− 0.16 (0.22)	0.15 (0.26)	0.10 (0.28)	− 0.88** (0.32)
**Education** (low)																
Secondary	0.61 (1.15)	0.03 (0.19)	0.11 (0.18)	− 0.15 (0.17)	0.12 (0.17)	0.04 (0.19)	− 0.03 (0.16)	0.00 (0.17)	− 0.18 (0.16)	− 0.25 (0.15)	− 0.15 (0.16)	0.02 (0.17)	0.08 (0.16)	− 0.24 (0.17)	− 0.20 (0.18)	− 0.36* (0.17)
Tertiary	− 0.60 (1.45)	− 0.08 (0.23)	0.01 (0.24)	− 0.21 (0.20)	− 0.13 (0.21)	0.03 (0.25)	− 0.33 (0.19)	− 0.18 (0.21)	− 0.28 (0.20)	− 0.35 (0.18)	− 0.22 (0.21)	− 0.22 (0.21)	− 0.28 (0.22)	− 0.66** (0.23)	− 0.41 (0.22)	− 0.26 (0.21)
*Make ends meet (easily)*																
Fairly easily	− 1.65 (1.35)	− 0.02 (0.16)	− 0.12 (0.16)	− 0.15 (0.15)	− 0.17 (0.15)	− 0.10 (0.14)	− 0.01 (0.14)	− 0.17 (0.16)	− 0.08 (0.15)	− 0.22 (0.15)	− 0.10 (0.16)	− 0.12 (0.15)	− 0.07 (0.15)	0.17 (0.14)	0.19 (0.15)	0.22 (0.15)
With difficulty	2.61 (1.61)	0.48* (0.24)	0.24 (0.23)	0.33 (0.20)	0.39 (0.23)	0.35* (0.24)	0.47* (0.19)	0.12 (0.20)	0.24 (0.23)	0.23 (0.22)	0.28 (0.20)	0.21 (0.23)	0.21 (0.22)	0.52* (0.22)	0.04 (0.21)	− 0.13 (0.20)
**Employed** (no)																
Yes	− 0.18 (1.16)	− 0.17 (0.18)	− 0.04 (0.18)	0.05 (0.16)	0.10 (0.16)	− 0.06 (0.17)	− 0.11 (0.15)	0.02 (0.14)	0.07 (0.18)	0.08 (0.15)	− 0.09 (0.16)	− 0.05 (0.18)	− 0.14 (0.16)	0.04 (0.16)	− 0.09 (0.18)	− 0.30 (0.18)
**Living area** (urban)																
Rural	− 0.19 (1.13)	− 0.08 (0.16)	0.09 (0.16)	− 0.11 (0.15)	− 0.04 (0.14)	0.26 (0.14)	0.16 (0.14)	0.03 (0.13)	0.09 (0.14)	− 0.10 (0.14)	− 0.08 (0.14)	− 0.25 (0.15)	− 0.17 (0.14)	− 0.06 (0.13)	− 0.19 (0.14)	0.02 (0.16)
*Type of house (house)*																
Apartment	1.54 (1.09)	0.18 (0.15)	0.27 (0.16)	0.26* (0.13)	0.31* (0.13)	0.28 (0.15)	0.12 (0.14)	0.13 (0.13)	0.28 (0.14)	0.26 (0.13)	0.10 (0.13)	− 0.06 (0.16)	− 0.01 (0.14)	0.02 (0.12)	− 0.09 (0.15)	− 0.11 (0.16)
*Self-rated health (bad health)*																
Good health	0.05 (1.61)	0.10 (0.22)	− 0.00 (0.19)	0.01 (0.19)	− 0.06 (0.20)	0.03 (0.21)	− 0.14 (0.25)	0.00 (0.19)	− 0.04 (0.18)	− 0.08 (0.19)	− 0.02 (0.18)	0.15 (0.16)	0.02 (0.17)	0.29 (0.15)	− 0.08 (0.18)	0.41* (0.16)
Very good/excellent health	− 0.22 (1.66)	− 0.02 (0.21)	− 0.02 (0.20)	− 0.18 (0.19)	− 0.05 (0.20)	− 0.18 (0.22)	− 0.34 (0.23)	− 0.14 (0.20)	− 0.03 (0.18)	− 0.20 (0.19)	0.10 (0.20)	0.12 (0.18)	0.01 (0.19)	0.23 (0.18)	− 0.52** (0.19)	0.11 (0.17)
**Depression**																
Yes	0.08 (1.46)	− 0.07 (0.23)	− 0.01 (0.19)	− 0.10 (0.17)	− 0.08 (0.18)	0.16 (0.17)	− 0.12 (0.20)	0.24 (0.17)	0.24 (0.16)	− 0.01 (0.18)	0.20 (0.20)	− 0.07 (0.19)	0.11 (0.18)	0.42** (0.15)	− 0.02 (0.18)	− 0.38* (0.1)
Observations	529	529	529	529	529	529	529	529	529	529	529	529	529	529	529	529

Bold values denote statistical significance at * p < 0.05, ** p < 0.01 and *** p < 0.001

This table reports the weighted estimates from the order probit model for the statements on human-animal interactions. Point estimates and standards errors in parentheses have the following significance levels: * *p* < 0.05, ** *p* < 0.01, *** *p* < 0.001. Data from SHARE wave 7, release version: 7.1.1

## Discussion

The current study is the first to explore pet ownership in a representative study of Swiss residents aged 55 years and over. Pet ownership is relatively evenly distributed across different socio-demographic strata of the older population in Switzerland. However, the presence of a companion animal is significantly lower among adults aged 75 years and older, older adults living in urban areas, and those living in an apartment rather than a house. Overall, pet owners had rather positive attitudes toward owning a companion animal, with positive statements about pet ownership generating much higher levels of agreement on a five-point Likert scale than negative statements about owning a pet. Among the potential challenges of pet ownership studied here, pet ownership’s high perceived financial costs represented the most important concern of owning a companion animal among older adults in Switzerland. These financial concerns were especially prevalent in older pet owners with lower education levels and those who found it generally challenging to make ends meet.

Loneliness and social disconnectedness can have a significant impact on older adults (Hawkley and Cacioppo 2007; Holt-Lunstad et al. 2010; Stanley et al. 2014), and finding new strategies such as promoting pet ownership to reduce the negative impact of isolation on mental health among older adults may have large welfare effects (Barker and Wolen 2008). Older individuals with fewer close human ties may especially benefit from owning a pet, as single, divorced, or widowed individuals often choose pets as substitutes for the missing partnership (Archer 1997). We find stronger pet bonding in non-partnered pet owners. Pet owners without a partner were more likely to state that their pets gave them feelings of happiness. Pet bonding also seemed to be higher for older pet owners. These results are consistent with other studies showing pet ownership as compensation for the absence of human companionship (Zasloff and Kidd 1994) and reduce the prevalence of bad mood (Turner et al. 2003).

A sense of purpose coming from an animal companion may give older adults a new focus of interest outside of themselves (Edney 1995) and require lifestyle adaptations to the pets. These behavioral changes may help older adults stay more active and provide opportunities to improve their social lives (Wood et al. 2005). A high proportion of pet owners in our study reported having discussions with others about their pets. In this context, pets can be a good topic to start conversations and serve as icebreakers. However, we did not find strong agreements when asking pet owners if their pets helped them to engage with other people and whether their pets made them go outside more frequently or not. A potential explanation of this finding may be that we considered many different types of pets and that some of them, such as cats, may not be very likely to trigger interactions with other people. When restricting the sample to dog owners only, more than 67% agreed that their dogs helped them meet new people, and approximately 82% stated that their dogs made them go outside more frequently. Hence, the exact nature of potential benefits of pet ownership will likely depend on the type of pet. Moreover, even if adopting a pet seems like a plausible strategy to fight isolation, our data show that pet ownership is much lower in the oldest age group, even if older pet owners seem to have better perceptions and experiences with their pets. This finding suggests potential preference-based selection into pet ownership, possibly reflecting particular challenges of pet ownership at older ages. Such difficulties may arise related to physical health, transportation, housing limitations, or other restrictions (Chur-Hansen et al. 2008). Another potential constraint to pet ownership is financial barriers. Older adults in our study were more inclined to agree that taking care of their pets was very costly. Therefore, although the results show positive perceptions and experiences of pet ownership among the oldest age group, increasing age and financial limitations can, in some cases, prevent the older old from adopting an animal companion.

We also found significant gender differences in human-animal interactions. Women seemed to be more attached to their pet(s), reporting more frequently than men that they loved their pets, enjoyed having them around, and considered them friends. These results are consistent with two other studies where women displayed a higher degree of attachment towards their pets (Martens et al. 2016; Simon and Nath 2004). Overall, women show greater empathy and a more positive attitude towards animals (Herzog 2007; Taylor and Signal 2005). Women in our study were also more likely to agree that they liked taking care of their pets and were more likely to do it alone. Compared to men, women tended to invest themselves more in the human-animal relationship. This finding is consistent with other studies that find that women report more frequently that pets reduce loneliness and help them get through hard times (Anderson et al. 2008). In our study, women indicated more strongly than men that their pets gave them a feeling of companionship and that living with a pet made them happier, potentially reducing loneliness.

The type of housing and the living area also matter for pet ownership, as they influence the kind of pets chosen and affect human-animal interactions (Poresky and Daniels 1998). In contrast to living in a house, owners sharing their lives with their pets in an apartment seemed to have better pet bonding and more benefits from pet ownership, which may also, at least in part, stem from preference-based selection into pet ownership. Respondents living in an apartment were more likely to report that their animals gave them companionship. They also seemed more connected to their pet as they had higher chances of agreeing that they loved taking care of their pets. Compared to living in a house, living in an apartment with pets tends to be associated with closer sharing of space, which may lead to closer interactions between pet owners and their animal companions (Poresky and Daniels 1998).

### Limitations

Our study suffers from several limitations. Some observations had to be dropped from the analysis due to missing data. However, the overall degree of missingness seemed relatively low (17,9%), and no critical patterns appeared when regressing an indicator for missingness on the set of covariates. A second potential issue concerns the definition of pet ownership (Adams et al. 2011). We consider all respondents who live in a household with a pet as pet owners. In practice, potential health-related benefits may be stronger for direct carers of pets (Smith 2012). Therefore, being able to assess the daily time spent with pets would have been a valuable extension of our analysis. Furthermore, other important details such as the age of the pets, the date of pet adoption, or the pet ownership history are also missing from the questionnaire, which could have provided additional important context for the relationships of pet owners with their pets. Finally, while our study presents new information on socio-demographic patterns in pet ownership and pet bonding among pet owners, our estimates cannot be interpreted as causal effects, as our data also suggest potentially important preference-based selection effects into pet ownership.

## Conclusion

Existing literature shows that having animal companions may benefit but also challenge their owners in various ways. Pets may help improve the general health of their owners, trigger social interactions, and add happiness to their owners’ everyday life. Still, they can also result in additional physical, mental, and financial burdens. Our study showed mostly positive perceptions and experiences of pet owners concerning their animal companions, highlighting that pets may play an important role in the healthy aging and well-being of older adults in Switzerland. Women had a higher level of attachment to their pets and also invested themselves more in the relationship. Although taking care of a pet can be demanding and requires many tasks, most older adults seem to have a healthy relationship with their pets. Pet owners without a partner were especially likely to report feelings of happiness from their pets. At the same time, the financial costs of pet ownership appeared to represent an important challenge for older pet owners, notably those with relatively low levels of education and more limited financial resources. Finally, the respondents’ accommodation was also associated with perceptions and experiences of pet ownership, as respondents who were living in a house were more inclined to have a pet. However, living in a flat was associated with better pet bonding, which may indicate important selection effects on pet ownership. In general, our findings suggest that pet ownership tends to improve the self-reported daily living in older adults who choose to own a pet. Further research including variables assessing individuals' social networks is necessary to understand better the causal impac of pet ownership on the well-being of older adults. Experimental, quasi-experimental, and longitudinal study designs could help evaluate the causal effects of human-animal interactions on the wellbeing of older adults.
